# Optimizing Anesthetic Selection in Transcatheter Aortic Valve Replacement: Striking a Delicate Balance between Efficacy and Minimal Intervention

**DOI:** 10.1155/2024/4217162

**Published:** 2024-02-29

**Authors:** Kahtan Fadah, Seyed Khalafi, Miller Corey, Jose Sotelo, Ahmed Farag, Tariq Siddiqui, Mehran Abolbashari

**Affiliations:** ^1^Division of Cardiology, Department of Internal Medicine, Texas Tech University Health Sciences Center, 4800 Alberta Avenue, El Paso, TX 79905, USA; ^2^Paul L. Foster School of Medicine, Texas Tech University Health Sciences Center, 5001 El Paso Drive, El Paso, TX 79905, USA; ^3^Department of Internal Medicine, Texas Tech University Health Sciences Center, 4800 Alberta Avenue, El Paso, TX 79905, USA; ^4^Tash Medical Clinic, 7812 Gateway Blvd E, El Paso, TX 79915, USA; ^5^Center of the Heart, 361 Vinton Rd, Vinton, TX 79821, USA

## Abstract

Patients with severe calcific native aortic valve stenosis (AS) who require valve replacement have two options, surgical aortic valve replacement (SAVR) or transcatheter aortic valve replacement (TAVR). TAVR was approved in late 2011 for extremely high-risk patients and was subsequently approved for high-risk (2012), intermediate-risk (2016), and low-risk (2019) patients. In 2019, TAVR procedures surpassed SAVR procedures for the first time in the United States. The approach to anesthesia for this procedure has also evolved. Initially, general anesthesia (GA) was preferred, but currently, conscious sedation (CS) is favored. This review aims to clarify the indications and contraindications for both approaches, as well as the advantages of one approach over the other. Recent studies show that conscious sedation has better outcomes in terms of all-cause mortality, procedure complications such as stroke, myocardial infarction, infection requiring antibiotics, acute kidney injury, and the need for inotropes or vasopressors.

## 1. Introduction

Transcatheter aortic valve replacement (TAVR) has revolutionized the field of cardiology in the 21st Century since the first procedure was successfully performed in 2002 by Cribier et al. [1, 2]. To date, a total of 300,000 procedures have been performed [3], and at least 300,000 more are expected to be performed annually by 2025 [4]. TAVR has become an alternative procedure for patients who were previously considered inoperable for surgical aortic valve replacement (SAVR). Recent studies have reported more advantages of TAVR compared to SAVR, including shorter hospital stays, decreased procedural time, and lower 1-year mortality rates [5].

To be eligible for TAVR, certain criteria must be met. The 2020 American College of Cardiology Vascular Guidelines indicate that patients ≥65 years, preferred bioprosthetic valve placement, beneficial ratio of life expectancy to valve durability, extensive calcification of the ascending aorta, unsuitable/high risk for SAVR, previous cardiac surgery with at-risk coronary grafts, or previous chest irradiation are all indications for TAVR. Contraindications for TAVR include an estimated life expectancy <1 year, inadequate annular size, hemodynamic instability, irreversible severe left ventricular systolic dysfunction, inadequate vascular access, active endocarditis, left ventricular thrombus, plaques with mobile thrombi in the ascending aorta or arch, elevated risk of coronary ostium obstruction, and no improvement in quality after TAVR [6].

The TAVR procedure involves the following five sequential steps: access, valve crossing, balloon aortic valvuloplasty (BAV), valve implantation, and access closure. Anesthesia selection is also an important consideration. The two most commonly used approaches are sedation and general anesthesia (GA). While sedation or GA can be used for TAVR, only GA may be used for SAVR. Sedation is defined as decreased consciousness induced by drugs, but the patient is still able to respond to verbal commands [7]. Multiple terms have been used for sedation, including conscious sedation, local anesthesia, monitored anesthesia care, and procedural anesthesia with analgesics [6]. Currently, both techniques are used interchangeably according to the anesthesiologist's preference and training. The question of whether sedation or GA is superior for patients undergoing TAVR remains important to consider.

## 2. Evolution of Anesthesia in TAVR

The evolution of anesthesia types used for TAVR has progressed over the past two decades. In the early years of TAVR, GA with endotracheal intubation and invasive monitoring were commonly used, including arterial line placement, pulmonary artery catheter placement, and transesophageal echocardiography (TEE) [8–10]. The initial thought was to deliver anesthesia similarly to any other cardiovascular procedure or SAVR [10]. However, GA was associated with extended procedure time and anesthesia-related complications [11, 12].

Recent advancements in valve delivery technology, including a reduction in valve and catheter sizes and increased operator skill, have led to a decrease in procedure length and complications with TAVR [9]. As a result, less invasive sedation techniques have been considered, leading to a shift away from GA. Diagnostic studies such as TEE were commonly expected before TAVRs to identify aortic regurgitations and complications during the procedure, such as annular rupture or cardiac tamponade [13]. However, clinicians have become more comfortable with cardiac-computed tomography (CT) scans alone for preprocedural planning and to evaluate the aortic anatomy [14].

Data from the Transcatheter Valve Therapy (TVT) Registry from the Society of Thoracic Surgeons/American College of Cardiology demonstrated that by 2016, approximately 50% of TAVRs were performed with alternative sedation types other than GA [15]. Non-GA approaches have become the leading form of sedation due to their associated safety profile and the rapid advancement of TAVR procedures.

Multiple terms have been used as alternatives to GA, including “conscious sedation,” “light sedation,” “moderate sedation,” and “procedural sedation,” all referring to the same approach. These techniques refer to the use of medications and maneuvers performed to help patients tolerate unpleasant or painful procedures and avoid potential unwanted memories associated with such procedures. The proper use of procedural sedation also aims to decrease the patient's perception of pain and is generally obtained through the administration of analgesics combined with a sedative [12, 16]. Conscious sedation, as defined by the American Society of Anesthesia (ASA), is a drug-induced depression of consciousness where patients can respond to verbal commands, do not require a patent airway, and maintain cardiovascular function [17]. The most commonly used medications during conscious sedation include propofol, midazolam, fentanyl, and other adjuvants [18]. Conscious sedation has also been referred to as moderate sedation but has been mostly replaced by the term “procedural sedation” [15].

The common characteristic of non-GA techniques is no requirement for endotracheal intubation, except for a few studies examining supraglottic airways for GA [19, 20]. Standardization has been described by the ASA, where the sedation should be referred to as monitored anesthesia care (MAC) when an anesthesiologist is present for cardiopulmonary monitoring, even if no sedative is administered [21]. Conversely, when an anesthesiologist is not present and the sedative medications are administered by a nurse on command by the TAVR operator, it is known as a “minimalist approach” (MA) [12].

### 2.1. Comparison of General Anesthesia and Conscious Sedation

Studies have retrospectively compared sedation methods with GA and have indicated potential benefits of conscious sedation such as lower rates of 30-day mortality, stroke, and shorter hospital stays for the non-GA groups [22]. Butala et al. conducted a study on 120,080 patients who underwent TAVR procedures from 559 sites and reported that the proportion of sites that used conscious sedation increased from 50% to 76% during the study [15]. The study found that the in-hospital mortality rate in patients who underwent conscious sedation was significantly lower than those who underwent GA (1.1% vs. 1.3%; adjusted RD: 0.2%; 95% CI: 0.4%–0.0%; *p*=0.010). Furthermore, the study noted that hospital length of stay for conscious sedation was significantly shorter (adjusted difference: 0.7 days; 95% CI: 0.8–0.7 days; *p* < 0.001).

Another study by Husser et al. with 16,543 patients from the German Aortic Valve Registry also found that conscious sedation had a lower thirty-day mortality rate compared to GA (3.5% vs. 4.9%; hazard ratio (HR): 0.72; 95% CI: 0.60–0.86; and *p* < 0.001) [23]. However, there was no difference in one-year mortality between the two groups (16.5% vs. 16.9%; HR: 0.93; 95% CI: 0.85–1.02; *p*=0.140) or in neurological dysfunction (2.8% vs. 2.9%; *p*=0.76). In addition, conscious sedation resulted in significantly shorter hospital stays and shorter ICU stays, with a higher proportion of cases with ≤1 day (38% vs. 34%, *p*=0.003) and a lower proportion of cases with ≥4 days (19% vs. 22%; *p*=0.001).

Oguri et al. conducted a study on 2,326 patients undergoing TAVR procedures from the French Aortic National CoreValve and Edwards 2 Registry and found no significant difference in thirty-day survival rate between conscious sedation and GA (89.3% versus 91.4%; *p*=0.27) as well as in one-year survival rates (77.7% versus 75.7%; *p*=0.44). Furthermore, there was no difference in stroke symptoms or length of hospital stay between the two groups [24]. Yamamoto et al. presented on 182 patients undergoing a TAVR procedure, with 130 undergoing conscious sedation. The study found no significant difference in mortality rate and stroke symptoms; however, there was a difference in length of hospital stay between the groups (8.1 ± 6.5 versus 12.2 ± 8.3; *p*=0.001) [25].

Authors of studies supporting conscious sedation suggest that the mortality benefits may be due to periprocedural neurological assessment, less vasopressor use, decreased procedure time, shorter ICU stays, and faster ambulation times [15]. However, some studies do not observe a difference between conscious sedation and GA, and further research is needed to confirm the complete switch. In addition, conscious sedation may be contraindicated in some instances, making GA the necessary plan of action.

### 2.2. When is Conscious Sedation Contraindicated?

Previous research indicates that conscious sedation is often preferred over general anesthesia, except in certain cases where conscious sedation is contraindicated. For example, patients who require real-time TEE monitoring during a TAVR procedure are better suited for general anesthesia due to minimal movement, which allows for monitoring for paravalvular leakage [23]. In contrast, patients under conscious sedation are more likely to have paravalvular leakage and misplaced valves during TAVR procedures due to increased movement. In addition, TEE monitoring is more commonly used during general anesthesia compared to conscious sedation, adding to the precision of the procedure [23].

General anesthesia is also preferred for patients undergoing subclavian or transapical TAVR approaches, as these approaches have a higher risk of complications, such as lung injuries and cardiac tamponade, and require limited movement during the procedure.

However, there is a concern regarding the periprocedural anxiety associated with conscious sedation, which may worsen patient movement. Studies have reported perioperative pain with conscious sedation, with a wide range of severity, but patients under general anesthesia are better able to control pain and manipulate the patient's position [11, 26].

If conscious sedation is chosen, anesthesiologists have the option to convert to general anesthesia if complications arise, but this conversion has been associated with increased risk of morbidity and mortality [15]. Therefore, centers with high rates of general anesthesia conversion may want to consider using general anesthesia instead of conscious sedation [27].  Table 1 presents an overview of the reviewed studies comparing general anesthesia and conscious sedation.

Conscious sedation has also been associated with ventilation depression and worsening of pulmonary arterial hypertension, so it is not ideal for patients who are elderly or have obstructive sleep apnea [26, 27].

### 2.3. Minimalist Approach

Some have questioned the necessity of having an anesthesiologist present during TAVR procedures. A study in Israel compared the outcomes of having an anesthesiologist perform MAC versus having the interventional cardiologist perform conscious sedation or MAC [30]. The study showed no significant difference in short-term outcomes, including adverse events and the length of hospitalization. This suggests that TAVR procedures can be performed without an anesthesiologist's assistance.

Most studies that have looked at anesthesia for TAVRs have defined general anesthesia (GA) to include endotracheal intubation for airway management. However, some have proposed using GA with a supraglottic airway (SGA) instead of an endotracheal tube for airway control. GA with ETT has been considered necessary to facilitate transesophageal echocardiography during TAVR, but new technologies have made SGAs effective for this purpose and other cardiac procedures [11, 31].

Azad et al. found that using SGA did not increase adverse outcomes and reduced operating room time and GA time [20]. In addition, patients in the SGA group received a lower number of opioids during the procedure and had a shorter length of stay. SGAs have been used in other procedures and offer shorter recovery times and possible decreases in the usage of inotropic and vasopressor agents that make GA more prone to intraoperative complications versus sedation techniques [20, 27].

A retrospective study compared SGA to MAC and found that SGA did not increase adverse effects [19]. This is important for patients with comorbidities who are not candidates for MAC and other less invasive sedation techniques. Both studies examining the potential role of SGA in TAVR were retrospective and limited to a single institution. However, SGA should be considered when GA is deemed essential for high-risk patients undergoing TAVR and possibly even for lower-risk patients as an intermediate approach [19, 20]. These studies support the use of SGA without increased harmful adverse effects compared to MAC.

### 2.4. Pharmacologic Choices in Non-GA Sedation

Pharmacologic choices for conscious sedation during TAVR have included a variety of agents, such as remifentanil, ketamine, propofol, midazolam, nalbuphine, and dexmedetomidine [32]. Studies have combined these agents in various ways, including ketamine with propofol [33], midazolam with nalbuphine [27, 34], ketamine with propofol or dexmedetomidine [32], midazolam with remifentanil [35], and propofol with remifentanil [25].

Behan et al. induced conscious sedation using remifentanil and found no difference in procedural time, postoperative outcomes, and length of hospital stay compared to general anesthesia [28]. Dehedin et al. used ketamine with as needed propofol for conscious sedation and found significantly decreased vasopressor use, creatinine elevation, and mortality rate compared to general anesthesia. However, there was no difference in left ventricular ejection fraction, procedural success, or nonlethal complications between the two groups [29].

Durand et al. used midazolam with nalbuphine for conscious sedation and found that periprocedural cardiopulmonary support was not required for any cases, with only 5 cases needing surgical intervention [34]. Ben-Dor et al. induced cases with ketamine with propofol or dexmedetomidine and found that 11.4% of the patients required conversion to general anesthesia due to respiratory failure or hemodynamic compromise [30]. Motloch et al. used midazolam with remifentanil and found that the only difference compared to the general anesthesia group was a lower glomerular filtration rate perioperatively [31]. Yamamoto et al. reported propofol with remifentanil conscious sedation, with 4.6% of the patients requiring conversion to general anesthesia due to vascular complications requiring operative therapy [25]. Finally, Park et al. used dexmedetomidine for conscious sedation and found no hemodynamic instability, no conversion to general anesthesia, and proper valve positioning [36].

Although there is no clear answer as to which sedative combination is best or at what dosing, most authors suggest that anesthesia medication is at the discretion of the anesthesiologist and hospital protocol.

A study comparing propofol and dexmedetomidine directly during MAC for TAVR found no difference in in-hospital and 30-day outcomes despite the two agents having different mechanisms of action that suggest likely differences in outcomes, particularly because of the profound cardiorespiratory depression associated with propofol. There was also no difference in vasoactive support during procedures, which was surprising as propofol was expected to require more. Despite the documented bradyarrhythmia that dexmedetomidine can induce, there was no difference in the onset of new arrhythmias between the two groups. There was also no difference in intraoperative vascular and bleeding complications between the two groups despite concerns regarding the sedation provided by dexmedetomidine. Another unexpected finding was that there was no difference in cost between the two agents despite dexmedetomidine having a higher shelf cost. This may have been due to the similar average hospital length of stay and ICU length of stay. Overall, the study's findings suggest that providers can use either agent comfortably as they tailor an anesthetic plan for their patient's TAVR [32].

Ketamine was studied as an adjunct during MAC in a retrospective study at a single center and was found to be associated with a reduction in 30-day mortality and cardiac arrest in the ketamine group [33].  Table 2 provides a summary of the reviewed studies, offering a comparison of pharmacologic choices for conscious sedation.

### 2.5. Future Directions

Although two prospective studies have been examined on the use of GA versus MAC or sedation, more prospective studies are needed to evaluate long-term effects. To gain insight into current methodologies and approaches, a survey for cardiac surgeons, cardiac anesthesiologists, and cardiologists could be developed and administered. Future studies should also aim to compare pharmacotherapy strategies to optimize the effectiveness of sedation for this specific procedure. The role of the anesthesiologist during the TAVR procedure has been reevaluated with the evaluation of the MA approach in the hospital, but its suitability requires further evaluation by the anesthesiologist [27].

Another possible approach under evaluation is the necessity of TEE for TAVR. Some argue that even trace amounts of paravalvular regurgitation in young patients can be detrimental and thus require TEE under general anesthesia [37]. However, more studies are needed to confirm this and whether it is actually a detriment. Many studies on TAVR anesthesia do not provide in-depth analysis of anesthetic approaches, so future studies on different approaches could help with morbidity and mortality benefits for patients undergoing TAVR.

An interesting question that could benefit the literature regarding this procedure is the use of remifentanil versus other opioids. Future research also needs to clarify the nomenclature when referring to sedation so that it is clear whether an anesthesiologist is present or not. The TVT Registry could incorporate the differences between MAC and the MA to facilitate a better understanding of these methodologies. Lastly, as new technology becomes available for device access and implantation, these topics should be restudied to ensure that patients are given the safest and most cost-effective anesthesia care possible for their procedure.

## 3. Conclusions

The evolution of anesthetic techniques for the TAVR procedure has shifted from general anesthesia towards conscious sedation over the last decade. Noninvasive techniques such as MAC, SGA, and the minimalist approach have been shown to have favorable short-term outcomes, with safer profiles and similar efficacy compared to GA. Therefore, conscious sedation is usually preferred over GA for TAVR due to its advantages in terms of reduced morbidity and mortality. However, a multidisciplinary approach should be considered when selecting the appropriate anesthetic form for patients with severe comorbidities and increased risk of complications. Future studies should aim to compare different pharmacotherapy strategies to optimize sedation effectiveness and to clarify nomenclature when referring to sedation. Overall, conscious sedation is the superior option for TAVR, and efforts should be made to promote its implementation and standardization in clinical practice.

## Figures and Tables

**Table 1:** Overview of the reviewed sources comparing general anesthesia and conscious sedation.

Authors	Year	Location	Purpose	Type of source	Summary points
Behan et al. [28]	2008	UK	Comparing general anesthesia and conscious sedation in TAVR procedures	Prospective cohort study	(i) No statistical difference in ICU transfer, procedure duration, and hospital stay between groups

Dehédin et al. [29]	2011	France	Comparing general anesthesia and local/regional anesthesia in TAVR procedures	Retrospective cohort study	(i) Incidence of hemodynamic instability and blood pressure required maintenance lower in the local anesthesia group
					(ii) No difference in procedural success and mortality rates in both groups

Ben-Doret al. [30]	2012	USA	Comparing monitored anesthesia care and general anesthesia in TAVR procedures	Prospective cohort study	(i) Procedure duration was significantly shorter for the MAC group
					(ii) Median intensive care unit and hospital stays were lower in the MAC group
					(iii) There was no significant difference in procedural complications between the groups
					(iv) Rise in creatinine was higher in the general anesthesia group

Motloch et al. [31]	2012	Austria	Comparing local anesthesia and general anesthesia in TAVR procedures	Retrospective cohort study	(i) Hemodynamic parameters were statistically more stable in the local anesthesia group
					(ii) Procedural time was significantly lower in the local anesthesia group
					(iii) Peak-systolic central aortic pressure and mean central aortic pressure were significantly higher in the local anesthesia group
					(iv) The need for adrenergic support during intervention was significantly lower in the local anesthesia group

Yamamoto et al. [25]	2013	France	Comparing general anesthesia and conscious sedation in TAVR procedures	Prospective cohort study	(i) No significant difference in procedural complications was seen between the groups
					(ii) The ejection fraction was significantly lower in the general anesthesia group
					(iii) The mortality predictive score was significantly higher in the general anesthesia group
					(iv) Total procedure time, length of hospital stay, and length of intensive care unit stay were significantly longer in the general anesthesia group

Oguri et al. [24]	2014	France	Comparing general and local anesthesia in TAVR procedures	Retrospective cohort study	(i) Cumulative 30-day mortality rates were significantly higher in the general anesthesia group, but no significant difference with propensity matching between the groups
					(ii) Hospital duration was significantly higher in the local anesthesia group
					(iii) No significant difference in postoperative complications between the groups

Mayr et al. [18]	2016	Germany	Comparing sedation and general anesthesia in TAVR procedures	Single-center, controlled, parallel-groupstudy with balanced randomization (1 : 1)	(i) No statistical difference in cerebral oxygenation or neurocognitive outcome
					(ii) Incidence of adverse events, specifically pain and respiratory depression were higher in the sedation group

Gurevich et al. [22]	2017	USA	Comparing minimalist approach to the standard TAVR procedure	Retrospective cohort study	(i) A minimalist approach was associated with improved procedural efficiency and reduced hospital length of stay
					(ii) No statistical differences in procedural or clinical outcomes was noted between the groups

Husser et al. [23]	2017	USA/Europe	Comparing general anesthesia and conscious sedation in TAVR procedures	Retrospective cohort study	(i) Fewer postprocedural outcomes were reported in the conscious sedation group

Butala et al. [15]	2020	USA	Comparing general anesthesia and conscious sedation in TAVR procedures	Retrospective cohort study	(i) Conscious sedation was associated with a decrease in in-hospital mortality, lower 30-day mortality, less use of inotropic drugs, shorter length of hospital stay, and more frequent discharges

Thiele et al. [10]	2020	Europe	Comparing general anesthesia and conscious sedation in TAVR procedures	Randomized clinical trial	(i) There was no statistical difference in all-cause mortality, stroke, myocardial infarction, infection requiring antibiotic treatment, and acute kidney injury between the groups
					(ii) No statistical differences in the median length of ICU and hospital stay were noted between groups
					(iii) There was no significant difference in device time, total procedural time, occurrence of delirium, moderate or severe prosthetic valve regurgitation at 30 days, ICU stay, hospital stay, and cardiovascular mortality between the groups
					(iv) The need and dose of vasopressors and inotropes were statistically higher in the general anesthesia group

Musuku et al. [19]	2021	USA	Comparing general anesthesia using a subglottic airway and monitored anesthesia care in TAVR procedures	Retrospective cohort study	(i) There was no significant difference between general anesthesia using subglottic airway and monitored anesthesia care in morbidity, 30-day mortality, and hospital or postanesthesia care unit length of stay

**Table 2:** Overview of the reviewed studies comparing the pharmacologic choices for conscious sedation.

Authors	Year	Location	Purpose	Type of source	Summary points
Kronfli et al. [32]	2021	USA	Comparing dexmedetomidine and propofol in TAVR procedures	Retrospective cohort study	(i) There was no significant differences in in-hospital outcomes, 30-day outcomes, or total cost of the patient's hospitalization between the groups

Zhao et al. [33]	2022	USA	Comparing ketamine and nonketamine drugs in TAVR procedures	Retrospective cohort study	(i) Ketamine had statistically lower 30-day mortality and lower intraoperative cardiac arrest
					(ii) There was no statistical difference in postoperative stroke, intraoperative conversion to general anesthesia, postoperative delirium, need for permanent pacemaker implantation, perivalvular leak, and length of stay between the groups

## Data Availability

No data were used to support the findings of this study.
